# A Pilot Study of a Creative Bonding Intervention to Promote Nursing Students' Attitudes towards Taking Care of Older People

**DOI:** 10.1155/2011/537634

**Published:** 2011-09-25

**Authors:** Ann R. Lamet, Rosanne Sonshine, Sandra M. Walsh, David Molnar, Sharon Rafalko

**Affiliations:** Division of Nursing, College of Health Sciences, Barry University, 11300 NE Second Avenue, Miami Shores, FL 33161-6695, USA

## Abstract

Although numbers of older people are increasing, nursing students have negative attitudes towards older people and do not plan to care for them following graduation. Multiple strategies have been implemented to reverse students' attitudes with mixed results. The purpose of this pilot quasi-experimental study was to test a Creative-Bonding Intervention (CBI) with students implementing art activities with older people to promote students' willingness to take care of them. Using a self-transcendence conceptual framework, control (*n* = 56) and experimental (*n* = 14) student groups were pre- and post-tested on attitudes toward older people, self-transcendence, and willingness to serve. The CBI improved attitudes towards older people with negative attitudes significantly changed (*P* = .008) but with no significant differences on self-transcendence and willingness to serve. However, willingness to serve results approached significance (*P* = .08). The willingness measure (one question) should be expanded. Curricula changes that incorporate creative activities such as the CBI with larger and equal numbers in student groups and longitudinal follow up to determine long-term results after graduation are suggested.

## 1. Introduction

The pervasiveness of negative attitudes toward older people and lack of nurses' interest in caring for them has become increasingly problematic as numbers of older people continue to increase worldwide [1–3]. Amid the current nursing shortage, there is a growing demand for nurses to care for the health care needs of older people [3]. Thirty years ago, D. Feldbaum and M. Feldbaum [4] addressed the issue of ageism in a United States survey of undergraduate nursing students (*n* = 3,942) and registered nurses (RN's) (*n* = 5,300) to describe student and RN attitudes about caring for older people. They concluded that persons who were interested in caring for older people were older themselves (>55), were of minority status, and had ongoing contact with older people at home or at work [4]. They suggested strategies to increase nurses' interest in older people that included recruiting more minorities into nursing, increasing financial incentives to nurses caring for older people, and increasing gerontology content in curricula in schools of nursing [4]. In an attempt to increase nursing students' willingness to care for older people after graduation, we offered nursing students suggestions for creative bonding activities they could share with older people.

Since the D. Feldbaum and M. Feldbaum study [4], numerous approaches have addressed issues related to ageism; yet, negative attitudes about older people among nursing and other healthcare professional students persist [3, 5, 6]. Minorities and older health professional students and workers have been cited as groups that favored work with older people [4]. Researchers have more recently reported an inverse relationship between age of the caregiver and better attitudes among Hispanic social work students [6]. Additionally, when geriatric didactic content has been added to the nursing school curricula, there have been inconsistent results with little or no gain in knowledge, little or no attitude change, and little or no interest in caring for older people after graduation [2, 7–9]. In a study of curricula in four schools of nursing, the one school that had made major curricula changes with a focus on older people reported changes in attitudes of students towards older people [1]. Such changes were attributed to (1) the adoption of a “successful aging paradigm” framework throughout the curricula, (2) the provision of in-service education classes to build faculty expertise in gerontology, and (3) the institution of multiple student experiences with well elders living in the community. However, students' willingness to care for older people post-graduation was not evaluated. In two recent qualitative studies, medical students' attitudes towards older people did improve after year-long contact between medical students and community dwelling older adults [10, 11]. The older adults served as mentors/advisors with the medical students [10, 11]. Similar mentor-student programs in schools of nursing have not been reported.

Questions remain about attitudes of students in healthcare professions and college students in general. Attitudes and knowledge about older people have been described in undergraduate and graduate college students (*n* = 125) [5]. In these college students, frequency of communication with elders was positively correlated with better attitudes about older people [5]. Yet, student age and knowledge about aging had no relationship to students' attitudes about older people. Other researchers have measured frequency of communication, proximity to older people, and student anxiety about aging and reported no relationship between attitudes of college students (*n* = 107) and frequency of daily contact with older people [7]. Yet, those students who resided with older people had more anxiety about aging than students whose sole contact with older people was in a work environment [7]. In a survey of physician assistant students (*n* = 36), students who had weekly socialization with older people did have better attitudes towards older people than students who had yearly contact [12]. In a small group (*n* = 27) of nutrition students, 1/2 of the students (*n* = 13) that preferred working with older people also had better attitudes and previous frequent contact with older people [13].

Throughout the literature, knowledge about aging [7, 8], frequency of contact [10–13], minority status [4], age of a student or caregiver [4–6], type of older person with whom one communicates [7, 10, 11, 13], and implementation of curricula changes [2, 10, 11, 13] may or may not determine if future health professionals are willing to care for older people. Surprisingly, in a longitudinal study of baccalaureate nursing students, the introduction of additional knowledge about elders and increased student contact with elders decreased students' interest rather than increased their interest in caring for older people [2]. Because nurses are the largest health care work force that will provide care for elders [3], researchers continue to focus on ways to increase nursing students' willingness to care for older people after graduation.

## 2. Background Studies for the Current Study

Researchers have reported novel approaches to address the challenges surrounding nursing students' knowledge, attitudes, and willingness to care for older people after graduation. The theory of self-transcendence [14] was used as a conceptual framework in two studies [15, 16] to test a creative bonding intervention (CBI) to promote positive communication between elders and undergraduate nursing students in the USA [15] (a pilot study) and in Taiwan [16] (a prepost test quasi experimental study).

## 3. The Theory of Self-Transcendence and Related Studies

The theory of self-transcendence [14] guided the present study and provided the rationale to test the CBI. In the theory of self-transcendence, Reed [14] identified three major concepts: self-transcendence, vulnerability, and well-being. In the present study, older people and students were considered vulnerable persons, the CBI was implemented to “activate” self-transcendence, and well-being was an expected outcome. Reed described self-transcendence as a developmental trait that may be used by vulnerable populations. Self-transcendence has four dimensions: (1) intrapersonal self-transcendence—within self—(2) interpersonal self-transcendence—between self and others—(3) temporal self-transcendence—past, present, and future time—and (4) transpersonal self-transcendence—outside, of self, for example, spirituality. Reed discussed that in vulnerable persons, the introduction of creative activities might serve to promote self-transcendence and well-being. Self-transcendence was one outcome variable in the present study measured by Reed's self-transcendence scale (STS).

In previous studies [15, 16], the focus of the communication between the student and older person was on the interpersonal self-transcendence (between self and others) and temporal self-transcendence (present, past, and future time). By self-report following the CBI, students [15, 16] reported that they had “grown” personally after their encounters with older people (intrapersonal self-transcendence). During the CBI activities to promote temporal self-transcendence, students were encouraged to ask the older person to reminiscence about their favorite colors, favorite past experiences, or previous hobbies during the CBI activities [15, 16].

In the two previous studies [15, 16] students attitudes [17, 18] and self-transcendence [14] were measured before and after a one semester course, “Care of the Older Adult.” In the larger study [16], an experimental group of students (*n* = 100) were randomly assigned to implement the CBI while a control group of students (*n* = 100) implemented a friendly visit only. Students in the CBI group increased in self-transcendence (*P* < .06); however, there was significant improvement (*P* < .05) in students' attitudes towards older people [16]. Students in the CBI group also had significantly greater volunteer visits (*P* < .01) [15, 16].

## 4. Purpose of the Study

The purpose of the present study was to utilize the CBI to continue to investigate the relationship of students' attitudes toward older people, to describe self-transcendence, and to evaluate students' willingness to take care of older people. Researchers inferred that if self-transcendence increased and attitudes towards older people improved, students would increase their willingness to take care of older people after graduation. The research question was, “in a group of senior baccalaureate nursing students, will the CBI improve attitudes towards older people, self-transcendence, and willingness to take care of older people?” One question to “measure” willingness was added to the demographic data sheet. This question asked students what age group preference they anticipated caring for after graduation.

Hypotheses were (1) compared to the control group students who participate in the CBI will have better attitudes towards older people; (2) compared to the control group students who participate in the CBI will have an increase in self-transcendence; (3) compared to the control group, students who participate in the CBI will have an increase in willingness to take care of older people following graduation.

## 5. Methods

### 5.1. Settings/Participants

The collection of data occurred during the fall of 2008 and the spring of 2009 at a Catholic Southeastern Florida university. This pretest posttest descriptive cross-sectional design included students from junior and senior classes that served as controls; one section of seniors (*n* = 28) was the experimental group where the CBI was piloted. A convenience sample of two groups of junior students and one group of senior students with no exposure to the CBI served as the control group (*n* = 84). There was no exclusion criteria for any of the participants enrolled in select junior and senior nursing courses. Initially the researchers intended the study as descriptive only, thus the small sample and unequal size between groups. After the decision was made to pilot the CBI, the institutional review board (IRB) (ethics board) approved adding the intervention only in time to include the final group of seniors in the “Care of the Older Adult” course during their final six weeks before graduation. Therefore, the implementation of the CBI was limited to a final group of 14 seniors. Absences at pretesting or post-testing and timing (seniors getting ready to graduate) contributed to the small sample for the CBI group. Both the CBI group and the control group of seniors had clinical experiences with older well people living in the community. There was a focus on well people in the “Care of the Older Adult” course due to the literature that has supported the need for young persons to have experiences with well older people before these people become ill [1, 10, 11].

### 5.2. Materials/Tools/Instruments

The questionnaire packet completed by the participants was comprised of a sociodemographic survey that included age, ethnicity, family structure, interaction with older people, living arrangements regarding older relatives or grandparents, visitation with older people or grandparents, volunteerism with older people, preference among three age categories to work with after graduation, and willingness to take care of older people. Two instruments administered before and after intervention were the *Self-Transcendence Scale* [14] and *Attitudes toward Old People Scale *[17, 18]. Data were gathered the first and last days of classes. Data were collected anonymously with volunteers choosing their own identification numbers for pre- and postdata packets.

Reed,s* Self-Transcendence Scale *(STS) [14] measured self-transcendence. Participants were asked to indicate on a 4-point Likert scale how strongly they agreed with the 15 statements (1: not at all, to 4: equals very much), resulting in scores ranging from 15 to 60 with the higher scores indicating higher levels of self-transcendence. Reed reported an alpha coefficient of  .90 indicating high internal consistency for the scale [14].

Attitude towards older people was measured using the *Attitudes toward Old People Scale* [17, 18]. Participants were asked to indicate on a 7-point Likert scale how strongly they agreed or disagreed with the 22 items. Seventeen items were positively worded and responses were summed to create a positive attitude subscale. Five items were reverse worded and used as a negative attitude subscale.

### 5.3. Ethical Considerations

The protocol and method of obtaining informed consent were approved by the institutional review board (IRB) of the university where researchers were affiliated. Anonymity was protected as no names were on any information in the packets with a reminder on the instruments not to put names on any of the materials. There was no way to link student responses to an individual student. One group of senior students (*n* = 28) enrolled in the course, “Care of the Older Adult,” was exposed to the CBI as part of the course content. The control group of students were from volunteers of three groups of students from junior and senior classes (*n* = 84). All information obtained was anonymous; therefore, students were not informed of their own scores. The course instructors were not present when research packets were distributed. Researchers implemented the CBI during class time with the experimental group of senior students. Control group students completed research instruments but did not receive the CBI. 

Student participation did not influence any course grades as instructors did not know who completed research packets.

### 5.4. The Creative Bonding Intervention (CBI)

**Figure 1:**
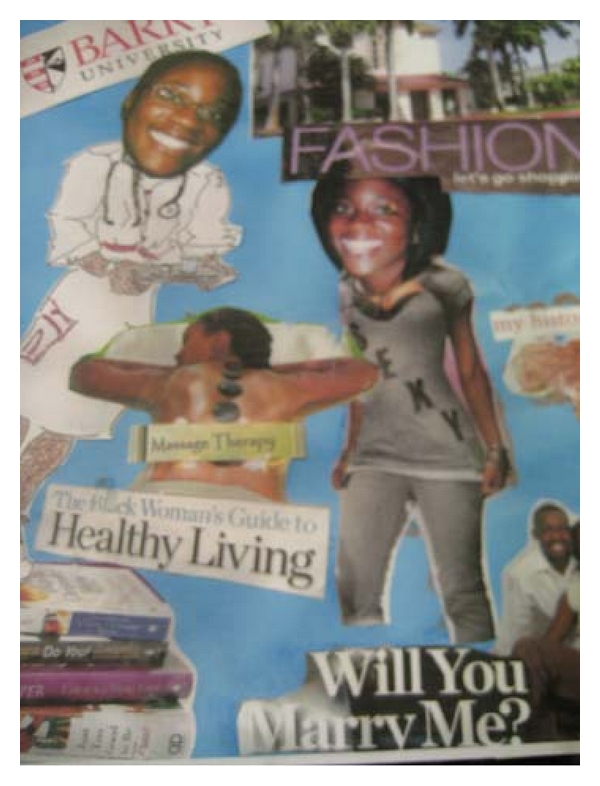


**Figure 2:**
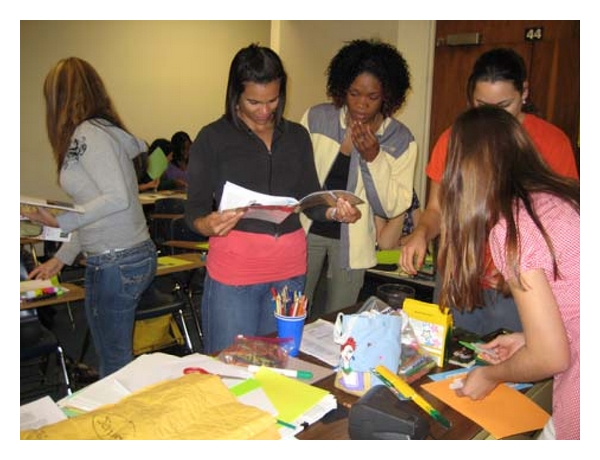


**Figure 3:**
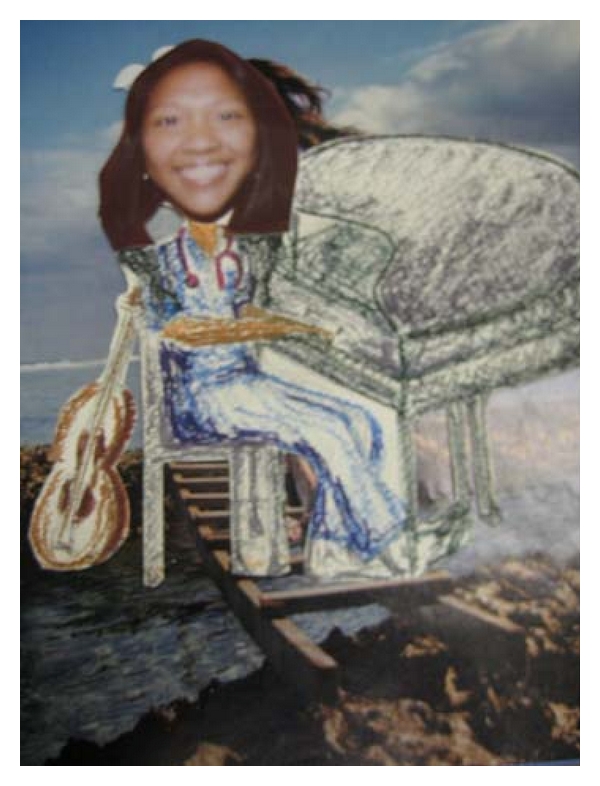


Two researcher “interventionists” who were not involved in teaching any didactic course content nor involved in student grading presented a mini-CBI one-hour workshop to the senior experimental students enrolled in the course, “Care of the Older Adult.” Students enrolled in the course were required to have 45 hours of clinical experience with older persons living in the community. The interventionists also offered additional time with interested students if students wanted more instruction about any of the three CBI activities. The CBI activities were (1) a monoprint, (2) a ribbon gem, and (3) the self-image portrait. Interventionists suggested that students might use their portrait to introduce themselves to individuals or groups of older people when they first met, following researchers' suggestions from previous CBI studies [15, 16] (see examples of photos of students completing the self-image activities and examples of student self-image portraits in Figures 1–3). If the older people were interested, students would offer them the opportunity to create their own self-portrait or create another CBI activity. A copy of the CBI workbook was given to each of the experimental students along with the needed art supplies to complete the three activities. The workbook that describes each activity can be obtained from the third author.

## 6. Results

Participants were 90% women and 70% minority with a median age of 25, including 14 in the intervention group and 56 in the control group. At some point in their lives half had worked or volunteered with older people and 44% lived with older relatives. The majority (57%) interacted with older people at least several times per week. 

Students in the control group were asked to identify their age group preference (children, middle-aged, or older people) to care for following graduation. Older people were the first preference at the pretest for 18% of the respondents; however, at the posttest only 9% of the respondents preferred to take care of older people. In contrast, the proportion of students that preferred the middle-aged remained constant at 50% and the proportion that preferred children increased from 33% to 41%. In the absence of the CBI, there was a dramatic decrease in preference to take care of older people, illustrating the problem the CBI was intended to address.

Although there were no statistically significant differences in baseline scores (see Table 1), the researchers used gain scores to protect against the possible confounding influence of baseline differences A one-tail independent-samples *t*-test was conducted to evaluate the hypothesis that students' positive attitudes toward older people would improve more for the CBI group (*M* = 10.71, SD = 11.09) than the control group (*M* = 3.79, SD = 11.74), *t*(68) = 1.99, *P* = .025. The standardized difference in means (*d* = .57) indicated a medium effect size. Students' negative attitudes toward older people improved more for the CBI group (*M* = −3.79, SD = 3.66) than the control group (*M* = −0.66, SD = 3.89), *t*(68) = 2.72, *P* = .008. The standardized difference in means (*d* = −.78) indicated a large effect size. On average, the CBI led to important improvement in attitudes toward older people, especially diminishing negative attitudes.

A one-tail independent samples *t*-test was conducted to evaluate the hypothesis that students' self-transcendence would increase more for the CBI group (*M* = .64, SD = 5.7) than the control group (*M* =  .59, SD = 6.17), *t*(68) =  .032, *P* = .49. A one-tail independent samples *t*-test was conducted to evaluate the hypothesis that students' willingness to take care of older people would increase more for the CBI group (*M* = 1.36, SD = 2.29) than the control group (*M* =  .22, SD = 2.37), *t*(58) = 1.45, *P* = .08. The rejection of this hypothesis was perhaps due to low power,  .30. Low power may be attributed to the small sample size, the unequal size of the groups, and imprecision in the willingness measure which was based on only one question. Although there was a positive correlation between willingness to take care of older people and positive attitudes, *r* = .24, *P* = .03, the increase in positive attitudes was not sufficient to generate a significant increase in willingness. It is important to note that willingness to take care of older people is uncorrelated with negative attitudes, *r* = −.06, *P* = .33.

## 7. Discussion

Even though this was a pilot study, limitations should be noted and corrected in future studies. First, the use of a convenience sample limited the generalizability of the results and introduced possible self-selection bias. Second, the glaring differences in sample size between the control group and the experimental group reduced the power of all the hypothesis tests. To increase numbers within the CBI group, phase II of the study is in progress implementing the CBI over two semesters with several classes of senior students enrolled in the “Care of the Older Adult” course. Better measurement of willingness to take care of older people should be developed. Finally, since differences in self-transcendence were not found on Reed's [14] STS, perhaps a different theoretical framework would better explain the effects of the CBI. Reed has acknowledged that self-transcendence changes may occur only over long periods of time.

Regardless of the small size of the CBI group, this pilot study provided evidence that the CBI improved students' attitudes towards older people, with increases in positive attitudes and even larger decreases in negative attitudes. Students often expressed surprise that the CBI experience was more enjoyable than they had expected. This experience seems to be more effective in destroying negative stereotypes than in building positive bonds. Perhaps positive bonding requires a longer timeframe. Although not statistically significant, there was evidence that the CBI promoted students' willingness to take care of older people after graduation. In the absence of the CBI there was a dramatic decrease in preference to take care of older people after graduation, The results suggest that interventions which promote positive attitudes toward older people are more effective in increasing willingness than interventions that disabuse students of the negative stereotypes that may underlie negative attitudes.

Given the shortage of nurses in settings that serve older persons such as skilled nursing facilities, the purpose of our study was to facilitate students' willingness to care for older persons after graduation. The course, “Care of the Older Adult,” was designed with a focus on well older persons. The expectation was that the CBI group would rethink their ideas about older persons if they had a positive “bonding” experience with well older people. In a previous study [16], when the CBI was implemented by students in a nursing home setting, similar results were achieved with improved attitudes towards older people.

In contrast to the previous literature [2, 7, 9] where there was little or no attitude change in students, our study found there were significant improvements in attitude in the CBI group. In previous studies [2, 7, 9] willingness to care for older people was not evaluated. Continued contact with community-dwelling older people among nursing students had a positive change in attitudes similar to the medical students [10, 11] when they had multiple contacts with community-dwelling well elders who served as mentors over a year-long period.

## 8. Conclusions

From our study and previous studies, multiple interventions with community-dwelling older people hold promise not only to change students' attitudes toward older people but also to promote students' commitment to care for an ever-growing older population. Due to the small number of experimental students in our study and only one question regarding willingness to take care of and preference for caring for specific age groups following graduation, additional questions are needed in these domains to discover new strategies to promote students' interest in older people. In the comparative study of four nursing schools [1], students in the school that adopted major curricula changes did have more positive attitudes, but students' plans after graduation were not reported.

Therefore, major curricula changes including creative activities such as the CBI and longitudinal follow up with students after graduation may provide educators with additional strategies to increase students' interest in older people. An intervention that allows nursing students to spend time with older people utilizing creative activities may provide a dual benefit. Students' interest in geriatric nursing may increase, and older people may benefit from the additional time students spend with them during implementation of creative activities.

## Figures and Tables

**Table 1:** Pre-test scores between groups.

	Experimental or control	*N*	Mean	Std. deviation	Std. error mean
Willingness to care score before intervention	Control	56	6.41	2.940	.393
	Experimental	27	6.41	2.500	.481
Positive attitudes toward elderly total score pre intervention	Control	57	75.21	11.437	1.515
	Experimental	27	77.15	9.980	1.921
Negative attitudes toward elderly total score before intervention	Control	57	15.39	4.913	.651
	Experimental	27	14.74	4.284	.824
Attitudes toward elderly total score before intervention	Control	57	59.82	13.503	1.788
	Experimental	27	62.41	11.369	2.188
Self-transcendence total score before intervention	Control	57	50.12	5.587	.740
	Experimental	27	49.04	4.895	.942
